# Detecting Introgressed Populations in the Iberian Endemic *Centaurea podospermifolia* through Genome Size

**DOI:** 10.3390/plants10081492

**Published:** 2021-07-21

**Authors:** Jaume Pellicer, Jordi López-Pujol, Marc Aixarch, Teresa Garnatje, Joan Vallès, Oriane Hidalgo

**Affiliations:** 1 Institut Botànic de Barcelona (IBB, CSIC-Ajuntament de Barcelona), Passeig del Migdia s.n., Parc de Montjuïc, Catalonia, 08038 Barcelona, Spain; jlopez@ibb.csic.es (J.L.-P.); tgarnatje@ibb.csic.es (T.G.); 2 Royal Botanic Gardens, Kew, Richmond TW9 3AE, UK; 3 c/Mossèn Manyà 15, Catalonia, 43500 Tortosa, Spain; marc_aixarch@hotmail.com; 4 Laboratori de Botànica, Unitat Associada al CSIC, Facultat de Farmàcia i Ciències de l’Alimentació, Institut de la Biodiversitat IRBio, Universitat de Barcelona, Av. Joan XXIII 27-31, Catalonia, 08028 Barcelona, Spain; joanvalles@ub.edu

**Keywords:** *Centaurea cephalariifolia*, *Centaurea podospermifolia*, *Centaurea* ×*loscosii*, conservation, genome size, hybridisation, introgression

## Abstract

Based on results from previous studies, populations of the Iberian endemic *Centaurea podospermifolia* north of the Ebro River are considered genetically pure, while those southward are introgressed, with genetic input from *C. cephalariifolia*. This phenomenon is particularly relevant, especially given both the endangered and protected status for the species, which can have consequences in how to best apply conservation strategies to maintain genetic resources in the species. The main goal of this study was to evaluate whether genome size assessments using flow cytometry can help distinguishing between pure, hybrid and introgressed populations, and hence become a powerful and cost-effective tool to complement comprehensive population genetic surveys. The results indicate that there are significant genome size differences between populations of *C. podospermifolia*, which are coincident with previous considerations of pure and introgressed populations. Given the simplicity and reproducibility of this technique, flow cytometry could become an effective tool for monitoring pure populations of this species and, indeed, become an integral part of the management plans that are mandatory for listed taxa.

## 1. Introduction

**Figure 1:**
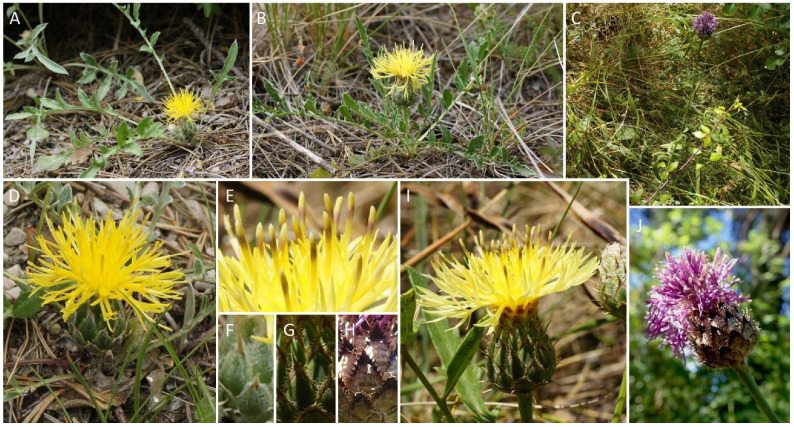
Photographs illustrative of the three *Centaurea* studied from Ports Massif, including morphological details of the flower head: (**A**,**D**,**F**) *C. podospermifolia*; (**B**,**E**,**G**,**I**) *C. ×loscosii*; (**C**,**H**,**J**) *C. cephalariifolia* (Images: Marc Aixarch, Oriane Hidalgo). Note that specimens of *C. podospermifolia* from Cardó Massif are morphologically no different from those of Ports Massif (image available at https://twitter.com/agentsruralscat/status/1278388147324243969?lang=ca, accessed on 19 July 2021).

About a decade ago, a study revealed that populations of the Iberian endemic *Centaurea podospermifolia* Loscos & J.Pardo north of the Ebro River (in the Cardó Massif) were considered genetically pure, while those from the south (in the Ports Massif) were introgressed, with genetic input from the widely distributed *C. cephalariifolia* Willk. [1]. Despite current understanding of the limitations of allozyme markers (e.g., low polymorphisms, restricted to coding genes), this study provided the first genetic insights into a potential hybridisation occurring between these two species, which had been based solely upon morphological evidence [2]. López-Pujol et al. [1] attributed this pattern to the existence of a biogeographical barrier, the Ebro River, that would have prevented the entry of genetic material of *C. cephalariifolia* into the Cardó Massif. Given that *C. podospermifolia* and *C.* ×*loscosii* Willk., the resulting hybrid between the two species are legally protected (listed as ‘vulnerable’ [3]), the question arose as to whether efforts should be geared towards conserving the so-called by the authors pure or hybrid populations of the species [1]. Conservation management strategies of endangered species require regular population monitoring. This is particularly important in cases such as that of *C. podospermifolia*, where hybridisation and introgression seem to be recurrent, and could possibly lead to the genetic assimilation of the species. Whilst *C. cephalariifolia* plants are large and vigorous, with purple florets and the involucre bracts with fimbriate margins (Figure 1C,H,J), *C. podospermifolia* and *C.* ×*loscosii* are morphologically more similar (Figure 1A,B), and mainly distinguished on the basis of flower head characters [2]. The main traits that can be used to distinguish between them are (i) the presence of sessile capitula in *C. podospermifolia*, while being pedunculate in *C.* ×*loscosii* (Figure 1A,B). (ii) Florets are bright yellow in the former and pale yellow (sometimes purplish), with darker anthers in the hybrid (Figure 1D,E,I). In addition (iii), involucral bracts in *C. podospermifolia* present mostly smooth margins and terminate with an apical spine (Figure 1F). In *C.* ×*loscosii*, the bracts also terminate in an apical spine, but the margins are clearly fimbriate (Figure 1G). Based on this, it is in many cases difficult to identify hybrid and introgressed individuals in the field, especially as few individuals flower per season, making identification in the field sometimes challenging [1].

Hybridisation is a recurrent phenomenon in plants, which has been widely recognised as a key evolutionary driver [4]. It has been linked in many cases to processes such as diversification and speciation [5,6] because of its role in the rise of new species through the accumulation of biological novelties inherent to the process [7,8]. Certainly, humans have benefited for long time from hybridisation by selecting and transferring useful traits among species for agro- and horticultural purposes. In many cases, hybridisation involves changes in chromosome number and/or ploidy level (e.g., *Medicago*, *Sorbus* [9,10]). Homoploid hybrids, where no change in ploidy with respect to the parental donors occurs, are also reported at lower frequencies among plants [11], though their rate of incidence is a hot topic of debate, and might have been so far underestimated [12].

**Figure 2:**
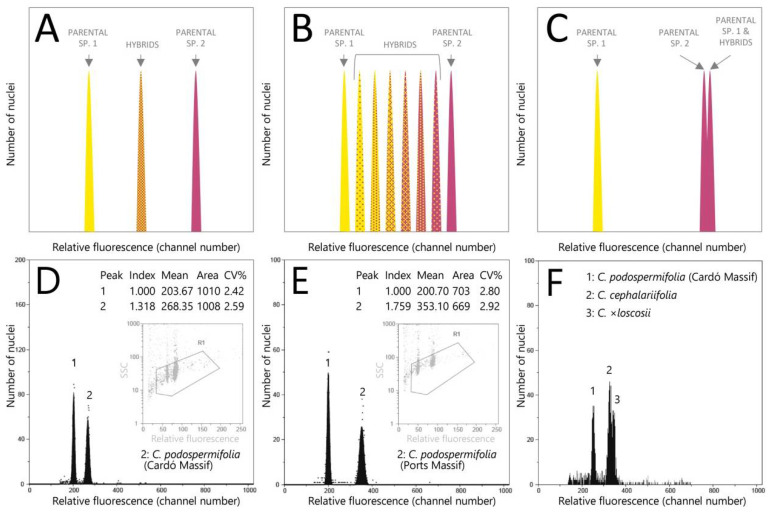
(**A**–**C**) Schematic flow cytometry outputs when processing together the parental species and their homoploid hybrid. (**A**) Configuration expected in case there is no gene flow between hybrids and parental species, due to, e.g., reproductive barriers or sterility of hybrids. (**B**) Configuration expected in case there is gene flow between hybrids and parental species. (**C**) Observed configuration in the studied populations. (**D**,**E**) Flow histograms obtained from analysing *C. podospermifolia* (peak 2) using *Petroselinum crispum* (4.5 pg/2C, peak 1) as the calibration standard. (**D**) Population from Cardó Massif (OH 621-1). (**E**) Population from Ports Massif (OH 618-4). (**F**) Flow histogram obtained from coprocessing *C. podospermifolia* (population from Cardó Massif, OH 621-4), *C. cephalariifolia* (OH 616-2) and *C.* ×*loscosii* (OH 613-5).

Besides the use of genetic markers to uncover the genetic signature of crosses between species, flow cytometry has been widely used at population level to detect changes in nuclear DNA contents, i.e., intraspecific and cytotype variation, which is frequently one of the outcomes of hybridisation (e.g., *Sorbus, Viola* [9,13,14]). Indeed, this technique has become a complementary tool to interpret output data from population genetics (i.e., allelic frequencies). To date, no genome size data are available for *C. podospermifolia*, *C. cephalariifolia* and their resulting hybrid *C.* ×*loscosii*, although we know they share a chromosome number of 2*n* = 40 [15], supporting that *C.* ×*loscosii* is a homoploid hybrid. With these premises in mind, the first question to address is whether nuclear DNA contents of *C. podospermifolia*, *C. cephalariifolia* and their hybrid are sufficiently different to be discriminated by flow cytometry. Theoretically, F1 hybrids are expected to have usually a genome size (or 2C-value) corresponding approximately to the mean of their parents (Figure 2A). Based on this, the present study aims at evaluating whether genome size assessments through flow cytometry could become a useful tool for population monitoring, offering a simple, cost-effective and high-throughput sampling method to assess the extent of hybridisation and introgression between these species. Several processes might account for the configuration depicted in Figure 2A, where all hybrids have a 2C-value that corresponds to the mean 2C of the parental species: (i) hybrids are infertile and are generated sporadically where the parental species coexist; (ii) hybrids are self-compatible; (iii) hybrids are apomictic, i.e., they produce seeds without sexual reproduction. In the case of self-compatibility or apomixis, one would therefore expect the hybrid to be stable (i.e., able to persist over time without genetic exchange with parent species).

Another question to answer is to which extent *C.* ×*loscosii* is a stable hybrid originating through one or few hybridisation events or if, by contrary, it is continuously generated in those areas where both parental species coexist (note that observations in the field tend to favour the latter hypothesis; J. López-Pujol and M. Aixarch, pers. obs.). In this sense, flow cytometry can also provide information on the different possible outcomes after generations of hybridisation and subsequent introgression, which would result in a diverse array of genome size patterns. For example, if a hybrid is fertile and there are no reproductive barriers with its parental species, then subsequent generations would have genome sizes that are not exactly the mean of the genome sizes of their parental species, but a range of intermediate values depending on the degree of introgression (Figure 2B).

## 2. Results

Flow cytometry results are given in Table 1 (mean values per population) and in Table S1 (values for each individual).

**Figure 3:**
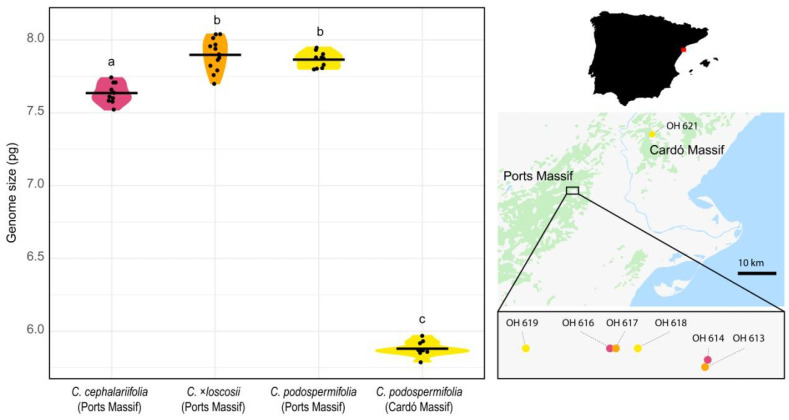
(**Left**) Violin plots showing the distribution of genome size in *Centaurea cephalariifolia*, *C.* ×*loscosii* and *C. podospermifolia* from Ports Massif and in *C. podospermifolia* from Cardó Massif. Horizontal lines represent the mean and dots indicate the genome size values of each individual. Letters above violins indicate which groups are statistically different from one another (Tukey test, *p* < 0.001). (**Right**) Location of the sampled populations of *C. cephalariifolia* (pink), *C.* ×*loscosii* (orange) and *C. podospermifolia* (yellow) in Catalonia, NE Spain.

We found *C. podospermifolia* population from Cardó Massif to have an average genome size of 5.88 pg/2C (Figure 2D), while populations of the same species from Ports Massif have a mean value of 7.87 pg/2C (Figure 2E). In fact, the three *Centaurea* species from Ports Massif exhibited very similar genome sizes (Figure 3), although that of *C. cephalariifolia* (7.64 pg/2C) was slightly smaller than that of *C.* ×*loscosii* (7.89 pg/2C) and *C. podospermifolia* (7.87 pg/2C).

To confirm that this variation was genuine rather than due to a potential technical artefact, leaves from populations showing the largest difference in 2C-values were mixed and processed. This resulted in distinct peaks in the flow histogram (Figure 2F), indicative of biologically real differences in nuclear DNA contents. In line with these findings, the Tukey test evidenced statistically significant differences in genome size between *C. cephalariifolia*, *C.* ×*loscosii* plus *C. podospermifolia* from Ports Massif, and *C. podospermifolia* from Cardó Massif (Figure 3).

## 3. Discussion

### 3.1. Genome Size Enables Discrimination between Populations of C. podospermifolia

This study reports novel genome size data, which were missing for the species and its hybrid. The analysis of nuclear DNA contents in multiple accessions of *C. podospermifolia* highlighted significant differences in genome size between the populations from Cardó and Ports Massifs, which covers the main distribution area of the species. Despite the limitations highlighted in the introduction regarding the use of the allozyme analysis for population genetics, the differences in genome size observed here can be interpreted considering the work by López-Pujol et al. [1], who reported the existence of segregated genetic clusters, coincident with each of the mountain ranges. Certainly, based solely on nuclear DNA contents, it would be too speculative to conclude that *C. podospermifolia* from Cardó Massif is genetically pure, especially since we cannot discard ancient hybridisation events that might have happened in that area. However, we cannot overlook the fact that this population has a significant smaller genome (5.88 pg/2C) than those that coexist with *C. cephalariifolia* in the Ports Massif (7.85–7.88 pg/2C), and such results indicate that, at least, the populations from both mountain ranges have undergone contrasting evolutionary trajectories. There are old records indicating the presence of *C. cephalariifolia* in the Cardó Massif [16,17,18]; however, these records are far enough from the current populations nuclei of *C. podospermifolia*, and currently the species is barely present in the area (Rafel Curto, pers. comm). Based on this, hybridisation might be unlikely given the results supporting a lack of gene flow between both mountain ranges [1].

It is nonetheless important to bear in mind that *C. cephalariifolia* is widespread in the Iberian Peninsula, and could eventually spread in this area, meaning that efforts should be made to preserve the original genetic stock of *C. podospermifolia* from this area. Furthermore, *C. podospermifolia* is included in the Catalan national list of protected species [3], and thereupon the conservation strategies to include populations that are unlikely to have undergone hybridisation (at least recent episodes) must be guaranteed. The conservation of introgressed populations, however, could also be desirable because, as already noted in López-Pujol et al. [1], it can have beneficial effects for an endangered species. Furthermore, Thompson et al. [19] argued that the conservation strategy for Mediterranean plants should combine the protection of pure and mixed populations—the latter being the arena for adaptive evolution and speciation.

Overall, in order to contribute further to the conservation and monitoring of these species, future population genetics surveys would be desirable to tackle, as well as the potential taxonomic questions arising from the apparent recurrent introgression in Ports Massif. The use of high throughput approaches (e.g., RADseq [13,20]) has been crucial to untangle the complex backgrounds of hybridisation in plants. Such techniques combined with flow cytometry would certainly enable reaching more sound conclusions about the genetic background of *Centaurea* in this area, including the level of introgression within and between populations. Altogether, this information would ultimately help to apply more fine-scale conservation protocols addressed to preserve genetic clusters of interest.

### 3.2. Interspecific Hybridisation and Introgression: Impact on Genome Size

As highlighted above, hybridisation and introgression are recurrent in plants and have been frequently associated with the rise of new species, and the genus *Centaurea* is not an exception [21]. Understanding the ecological and evolutionary consequences of this puzzling phenomenon has therefore become a source of intense debate [12,22,23,24]. Part of such interest is underpinned by the fact that, despite the expectancy that hybrids would (theoretically) display intermediate genomic features concerning the parental genomes [25], several studies report that, in many cases, one of the parental genomes can become dominant and erode the signature of the other parental genome [26,27,28]. In this sense, the pattern found in *C. podospermifolia*, whose likely introgressed populations present genome sizes close to their sympatric congeners and quite divergent from the isolated conspecific population (Figure 1C), in a similar way than what was observed in *Armeria* [29], could reflect a process of genomic assimilation. In the case of *Onopordum*, though nuclear DNA contents of hybrid and introgressed specimens between *O. hinojense* and *O. nervosum* displayed a range of sizes that fell within that of the parental species [30], the authors provided compelling evidence of the impact of directional introgression on the extinction risk of the endangered species *O. hinojense*. In fact, in absence of reproductive barriers, high levels of introgression can compromise the long-term existence of parental donors through assimilation of the genome [31], showing significant outperformance of hybrids in contact zones [23].

In addition, hybridisation can also affect the composition of repetitive DNA content of hybrids (triggered by the ‘genomic shock’), such as is the case in *Helianthus*, where the expansion of repetitive elements resulted in the overall genome additivity observed in homoploid hybrids, although the influence of ecological conditions in promoting such patterns is unclear [32,33]. Whether the increased genome size of introgressed *C. podospermifolia* is a consequence of genomic assimilation, repetitive element activation or the combination of both, yet remains to be clarified with a more in-depth population genetics screening.

Our flow cytometry analyses revealed that populations of *C. podospermifolia* from Ports Massif present very homogenous genome size values, suggesting that their increased genome size likely arose through an ancient post-hybridisation genome restructuring event, which could be now fully stabilised. By contrast, *C.* ×*loscosii* hybrids displayed more heterogeneous genome size values compared to their progenitors, having in some cases slightly larger C-values than those of the potential parents (Figure 2; Table S1). A scenario where *C.* ×*loscosii* presents more unstable genome sizes could indeed reflect the potential impact of recurrent and recent hybridisations in those areas where both parental species currently coexist. Nonetheless, this point should be confirmed with future genetic screening at the population level. In summary, hybridisation is increasingly recognised as both a potential threat (through hybridisation swamping and genetic assimilation, or outbreeding depression), but also a chance (as it generates genetic variation) for species survival and conservation [24,34]. In this sense, improving our understanding of hybridisation processes is therefore fundamental for species management, including its impact on genome size, which is most often overlooked.

## 4. Materials and Methods

### 4.1. Plant Sampling

Details of the studied populations are given in Table 1 and their location is shown in Figure 2. In total, 43 individuals were sampled, representing two populations of each species from Ports Massif, and one population of *C. podospermifolia* from Cardó Massif. We collected one leaf per individual, since approximately only 1 cm^2^ of leaf material is necessary to carry out genome size assessments by flow cytometry [35]. Herbarium vouchers from previous collections in the same area by López-Pujol et al. [1] are deposited in herbarium BC (Botanical Institute of Barcelona).

### 4.2. Flow Cytometry Measurements

Genome size was determined using propidium iodide flow cytometry with a CyFlow Space (Sysmex-Partec, Germany), following the one-step procedure [36] with modifications as described in Clark et al. [37]. We used *Petroselinum crispum* (Mill.) Fuss. ‘Champion Moss Curled’ (2C = 4.50 pg [38]) as calibration standard and the general purpose buffer GPB [39] supplemented with 3% PVP-40 [35]. The number of individuals analysed per population is depicted in Table 1.

### 4.3. Statistical Analyses

Data visualisation and analyses were carried out with the R package ggplot2 [40] and R v.3.2.2 [41]. After verifying assumptions of homogeneity of variances (with Bartlett’s test) and of normality on residuals (with Shapiro test and QQ-plot), we proceeded to a one way ANOVA and performed multiple pairwise comparisons between the means of groups (Tukey honest significant differences, R function: ‘TukeyHSD’).

## 5. Conclusions

Understanding hybridisation and the impact of introgression in plant populations is fundamental for species management, especially when it comes to endangered species. Besides the critical role of population genetics, complementary approaches can be implemented, including changes on genome size, which are most often overlooked. This study provides an example about how can nuclear DNA contents highlight potential hybridisation. Because of its simplicity and low cost, flow cytometry could become an effective tool for the monitoring of pure (and, eventually, introgressed) populations and, indeed, an integral part of the management plan that is mandatory for listed species, as illustrated here in the case of *C. podospermifolia*.

## Figures and Tables

**Table 1:** Genome size data for populations of *Centaurea cephalariifolia*, *C.* ×*loscosii* and *C. podospermifolia* at Cardó and Ports Massifs (Catalonia, Spain), with indication of geographical coordinates.

Massif	*Centaurea* Species (Population)	2C in pg (SD) ^1^	N ^2^	CV_plt_^3^	CV_std_^4^	Geographical Coordinates
Ports	*C. cephalariifolia* (OH 614)	7.66 (0.05)	5	3.21	2.86	40°48′25″ N, 0°20′15″ E
	*C. cephalariifolia* (OH 616)	7.61 (0.08)	5	2.51	2.35	40°48′28″ N, 0°19′40″ E
	*C.* ×*loscosii* (OH 613)	7.94 (0.11)	8	2.79	2.61	40°48′23″ N, 0°20′14″ E
	*C.* ×*loscosii* (OH 617)	7.83 (0.06)	5	2.67	2.64	40°48′28″ N, 0°19′42″ E
	*C. podospermifolia* (OH 618)	7.85 (0.06)	5	2.70	2.85	40°48′40″ N, 0°19′50″ E
	*C. podospermifolia* (OH 619)	7.88 (0.04)	5	2.92	2.87	40°48′28″ N, 0°19′10″ E
Cardó	*C. podospermifolia* (OH 621)	5.88 (0.05)	10	2.91	2.84	40°56′28″ N, 0°35′06″ E

^1^ SD: standard deviation. ^2^ N: number of individuals measured. ^3^ CV_plt_: coefficient of variation for *Centaurea* accessions (in %). ^4^ CV_std_: coefficient of variation for the calibration standard (in %).

## Data Availability

The data presented in this study are available in Supplementary Materials Table S1.

## Supplementary Materials

The following are available online at https://www.mdpi.com/article/10.3390/plants10081492/s1, Table S1: Genome size data for each of the individuals of *Centaurea cephalariifolia*, *C.* ×*loscosii* and *C. podospermifolia* at Cardó and Ports Massifs (Catalonia, Spain).
