# Supplementary material for: Detecting Introgressed Populations in the Iberian Endemic Centaurea podospermifolia through Genome Size
Source: Plants (Basel). 2021 Jul 21;10(8):1492. doi: 10.3390/plants10081492 (PMC8401423; doi:10.3390/plants10081492)
Supplement: Supplementary file 1 [file plants-10-01492-s001.zip › plants-1288910-supplementary.pdf]

Table S1. Genome size data for each of the individuals of *Centaurea cephalariifolia*, *C.* ×*loscosii* and *C. podospermifolia* at Cardó and Ports Massifs (Catalonia, Spain).

Species	Population	Individual	2C (pg)	[1]SD	[2]CVstd	[3]CVplant
C. cephalariifolia	OH 614	1	7.60	0.00	2.36	2.41
C. cephalariifolia	OH 614	2	7.71	0.02	3.40	4.00
C. cephalariifolia	OH 614	3	7.66	0.00	2.71	2.94
C. cephalariifolia	OH 614	4	7.61	0.01	2.79	3.09
C. cephalariifolia	OH 614	5	7.71	0.01	3.04	3.61
C. cephalariifolia	OH 616	1	7.58	0.01	2.55	2.61
C. cephalariifolia	OH 616	2	7.64	0.00	2.39	2.63
C. cephalariifolia	OH 616	3	7.74	0.00	2.15	2.35
C. cephalariifolia	OH 616	4	7.52	0.00	2.12	2.42
C. cephalariifolia	OH 616	5	7.58	0.01	2.53	2.54
C. ×*loscosii*	OH 613	1	7.86	0.02	2.77	2.73
C. ×*loscosii*	OH 613	2	8.04	0.01	2.56	2.86
C. ×*loscosii*	OH 613	3	7.70	0.03	2.58	2.73
C. ×*loscosii*	OH 613	4	7.97	0.00	2.41	2.71
C. ×*loscosii*	OH 613	5	8.01	0.01	2.44	2.72
C. ×*loscosii*	OH 613	6	7.94	0.00	2.71	2.72
C. ×*loscosii*	OH 613	7	7.96	0.00	2.81	2.72
C. ×*loscosii*	OH 613	8	8.04	0.00	2.56	3.14
C. ×*loscosii*	OH 617	1	7.88	0.05	3.30	2.89
C. ×*loscosii*	OH 617	2	7.90	0.03	2.25	2.59
C. ×*loscosii*	OH 617	3	7.82	0.01	2.32	2.31
C. ×*loscosii*	OH 617	4	7.79	0.03	2.60	2.89
C. ×*loscosii*	OH 617	5	7.76	0.01	2.72	2.67
C. podospermifolia	OH 618	1	7.80	0.01	2.79	2.58
C. podospermifolia	OH 618	2	7.90	0.01	3.04	2.83
C. podospermifolia	OH 618	3	7.80	0.01	2.80	2.31
C. podospermifolia	OH 618	4	7.93	0.01	2.88	2.90
C. podospermifolia	OH 618	5	7.81	0.02	2.73	2.89
C. podospermifolia	OH 619	1	7.95	0.01	3.20	3.29
C. podospermifolia	OH 619	2	7.88	0.00	2.70	2.64
C. podospermifolia	OH 619	3	7.88	0.03	2.85	2.71
C. podospermifolia	OH 619	4	7.83	0.00	2.92	3.02
C. podospermifolia	OH 619	5	7.87	0.00	2.69	2.92
C. podospermifolia	OH 621	1	5.93	0.01	2.39	2.50
C. podospermifolia	OH 621	2	5.87	0.00	2.74	2.86
C. podospermifolia	OH 621	3	5.86	0.02	3.29	3.08
C. podospermifolia	OH 621	4	5.79	0.01	3.21	3.13
C. podospermifolia	OH 621	5	5.87	0.04	3.24	3.17
C. podospermifolia	OH 621	6	5.87	0.00	2.78	2.84
C. podospermifolia	OH 621	7	5.85	0.00	2.23	2.27
C. podospermifolia	OH 621	8	5.87	0.02	2.81	2.73
C. podospermifolia	OH 621	9	5.92	0.01	2.97	3.16
C. podospermifolia	OH 621	10	5.97	0.01	2.78	3.38

[1]SD: standard deviation. [2]CV_{std}: coefficient of variation of the standard (in %). [3]CV_{std}: coefficient of variation of the *Centaurea* samples (in %).
